# Aberrant *O*-GlcNAcylated Proteins: New Perspectives in Breast and Colorectal Cancer

**DOI:** 10.3389/fendo.2014.00193

**Published:** 2014-11-11

**Authors:** Parunya Chaiyawat, Pukkavadee Netsirisawan, Jisnuson Svasti, Voraratt Champattanachai

**Affiliations:** ^1^Applied Biological Sciences Program, Chulabhorn Graduate Institute, Bangkok, Thailand; ^2^Laboratory of Biochemistry, Chulabhorn Research Institute, Bangkok, Thailand

**Keywords:** breast cancer, cancer biomarker, colorectal cancer, hexosamine biosynthesis pathway, *O*-GlcNAcylation, phosphorylation

## Abstract

Increasing glucose consumption is thought to provide an evolutionary advantage to cancer cells. Alteration of glucose metabolism in cancer influences various important metabolic pathways including the hexosamine biosynthesis pathway (HBP), a relatively minor branch of glycolysis. Uridine diphosphate *N*-acetylglucosamine (UDP-GlcNAc), an end product of HBP, is a sugar substrate used for classical glycosylation and *O*-GlcNAcylation, a post-translational protein modification implicated in a wide range of effects on cellular functions. Emerging evidence reveals that certain cellular proteins are abnormally *O*-GlcNAc modified in many kinds of cancers, indicating *O*-GlcNAcylation is associated with malignancy. Since *O*-GlcNAc rapidly on and off modifies in a similar time scale as in phosphorylation and these modifications may occur on proteins at either on the same or adjacent sites, it suggests that both modifications can work to regulate the cellular signaling pathways. This review describes the metabolic shifts related to the HBP, which are commonly found in most cancers. It also describes *O*-GlcNAc modified proteins identified in primary breast and colorectal cancer, as well as in the related cancer cell lines. Moreover, we also discuss the potential use of aberrant *O*-GlcNAcylated proteins as novel biomarkers of cancer.

## Introduction

Glucose consumption is required by living cells. Through glycolysis, glucose is mainly broken down into pyruvate, which enters into the tricarboxylic acid (TCA) cycle for maximum energy production. Cancer cells, however, uptake glucose at a higher rate and produce lactic acid rather than metabolizing pyruvate through the TCA cycle. This adaptive metabolic shift is termed the Warburg effect (1), leading to anaerobic glycolysis, and is thought to provide an evolutionary advantage to cancer cells by providing both increase bioenergetics and biosynthesis (2). Many proto-oncogenes (e.g., Ras and Myc) and tumor suppressors (e.g., p53) influence metabolism, and mutations in these genes can upregulate glucose uptake in cancer cells and promote a metabolic phenotype supporting tumor cell growth and proliferation (3). Elevated glucose uptake in cancer cells can be applied to monitor the location of primary and metastatic tumor sites; for an example, using F-18 fluorodeoxyglucose (FDG), a glucose analog, with a combination of positron emission tomography/computed tomography (PET/CT) (4). In general, most glucose enters into the glycolytic pathway, but a small fraction of glucose goes to the hexosamine biosynthesis pathway (HBP). This pathway generates a nucleotide sugar, uridine diphosphate *N*-acetylglucosamine (UDP-GlcNAc), used for many reactions including a sugar donor, and in multiple glycosylation reactions such as proteoglycan synthesis, *N*-linked glycosylation, and the formation of *O-*linked glycoproteins or *O*-GlcNAcylation (5).

The *O*-GlcNAc is dynamically regulated by two key enzymes: *O*-GlcNAc transferase (OGT) (6) and *O*-GlcNAcase (OGA) (7), for the addition and removal of a single GlcNAc residue from proteins, respectively. Unlike classical glycosylation present in endoplasmic reticulum (ER) and golgi apparatus, *O*-GlcNAcylation takes place in the cytoplasm, nucleus, and mitochondria, and is implicated in a wide range of effects on cellular function and signaling in metabolic diseases and cancer (8). Almost three decades since its discovery, more than 1,000 *O*-GlcNAcylated proteins have now been identified (9). Growing evidence reveals that *O*-GlcNAcylation has extensive crosstalk with phosphorylation either on the same or adjacent sites of various proteins (10). The interplay of these two post-translational protein modifications (PTMs) can work to fine-tune the regulation of target protein functions, stabilization, translocation, complex formation, and enzyme activity, which subsequently affects cellular signaling pathways. Glucose flux into the HBP resulting in increased *O*-GlcNAcylation is an emerging paradigm of the integration of metabolic and signaling networks. This review emphasizes the recent connection between the HBP and metabolic shifts, *O*-GlcNAc cycling enzymes, and *O*-GlcNAcylation and phosphorylation found in most cancers. In addition, *O*-GlcNAcylated proteins have been identified in primary breast and colorectal cancer (CRC) as well as in their cancer cell lines. Moreover, *O*-GlcNAcylated proteins are discussed as potential candidates for novel biomarkers of cancer.

## HBP, Metabolic Shifts, and Cancer

**Figure 1:**
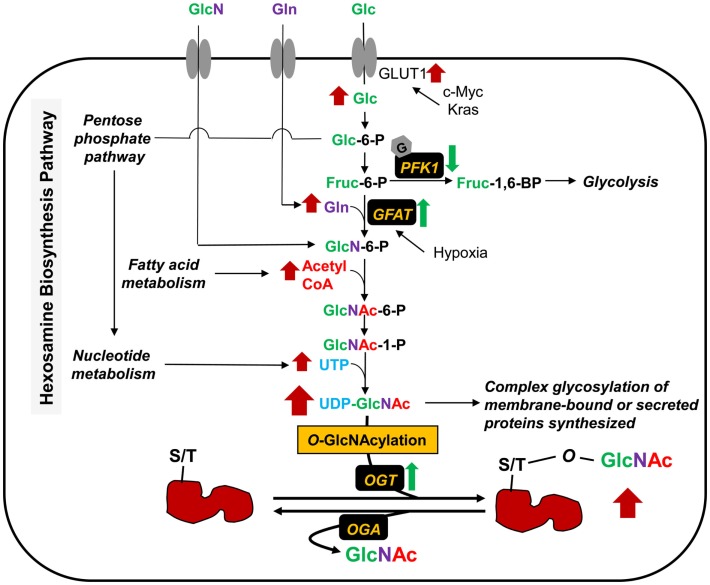
**Metabolic shifts through the hexosamine biosynthesis pathway (HBP) and protein *O*-GlcNAcylation in cancer**. The HBP produces UDP-GlcNAc from its parts, glucose (Glc), glutamine (Gln), acetyl-CoA, and UTP. The levels of these various metabolic inputs are all increased in cancer cells. Glucose is transported into cells by glucose transporters (e.g., GLUT1). Overexpression or mutation of *c-Myc* and *Kras* leads to an increase of glucose uptake through GLUT1 activation. *O*-GlcNAcylation of PFK1 suppresses the enzyme activity, resulting in redirection of glucose metabolism. GFAT is the rate-limiting enzyme for glucose entry into the HBP, which converts Fruc-6-P and glutamine (Gln) into GlcN-6-P. Hypoxia induces the GFAT transcription and expression. Glucosamine (GlcN) enters into cells via the glucose transporters and is phosphorylated to GlcN-6-P by hexokinase, bypassing GFAT. UDP-GlcNAc serves as a sugar donor of classical glycosylation and *O*-GlcNAcylation. The later glycosylation, taking place in cytoplasm, nucleus, and mitochondria, is controlled by *O*-GlcNAc cycling enzymes; OGT and OGA for the addition and removal of sugar in and out of proteins, respectively. OGT level is also upregulated, and consequently results in an increase of *O*-GlcNAcylation in several kinds of cancers.

The final end product of the HBP is UDP-GlcNAc (Figure 1), which is built up from glucose, glutamine, fatty acid (acetyl-CoA), and uridine. *O*-GlcNAc level may, therefore, be considered as a nutrient sensor for both normal physiology and disease pathophysiology, such as diabetes and cancer. Approximately 2–4% of glucose uptake into the cells enters into the HBP (11). Oncogenic genes (e.g., *c-Myc, Kras*) and hypoxia contribute in glucose metabolism. Osthus et al. showed that overexpression of c-Myc directly transactivates genes encoding glucose transporter GLUT1 and increases glucose uptake (12). Ying et al. also reported that activation of oncogenic Kirsten rat sarcoma viral oncogene homolog (Kras) is required in stimulating glucose uptake in an *in vivo* model of pancreatic ductal adenocarcinoma (PDAC) (13). Loss of Kras functions led to the downregulation of glucose uptake. Moreover, the levels of metabolites of the pentose phosphate pathway (PPP) and the HBP, as well as *O*-GlcNAcylation were decreased upon Kras inactivation. The rate-limiting enzyme in the HBP, glutamine:fructose-6-phosphate amidotransferase (GFAT) catalyzes the conversion of fructose-6-phosphate to glucosamine-6-phosphate. Hypoxia has been reported to induce the transcription of GFAT gene through the hypoxia responsive element (HRE) (14). Guillaumond et al. also showed that hypoxia increases the levels of GFAT mRNA expression and *O*-GlcNAcylation in pancreatic cancer cells (15). Blocking of GFAT activity by azaserine led to a decrease in hypoxic cell number, suggesting that activation of the HBP is required for survival of hypoxic pancreatic cancer cells. Glucosamine, although normally present at low levels in bodily fluids, enters into cells via the glucose transporters (16) and is phosphorylated to glucosamine-6-phosphate by hexokinase, thereby bypassing GFAT and elevating UDP-GlcNAc levels. Recently, Yang et al. demonstrated that radiolabeled glucosamine analogs can be introduced as novel agents to complement FDG imaging to increase specificity and improve the accuracy of lesion size in oncology applications (17). Glucosamine analogs become UDP-GlcNAc analogs and the newly modified *O*-GlcNAc analog proteins catalyzed by OGT are found in the cytoplasm and nucleus, whereas FDG, a glucose analog, is not metabolized and remains in the cytoplasm of cells. This method can be used to tag transcription factors known to be modified by *O*-GlcNAc (e.g., Sp1 and NF-κB), which are moved from the cytoplasm into the nucleus upon stimulation or activation in cancer cells. Glucosamine analogs can, therefore, be used in nuclear imaging to observe bio-activity in tumors.

In cancer cells, hyperglycemia is thought to feed the HBP and promote abnormally elevated *O*-GlcNAcylation of key signaling proteins (18, 19). Phosphofructokinase 1 (PFK1), one of the key enzymes in the glycolysis pathway, is modified by *O*-GlcNAc at Ser-529 in response to hypoxia in the lung cancer cell line, H1299 (20). This glycosylation suppressed PFK1 activity and redirected glucose flux through the PPP, thus increasing nucleotide metabolism and providing a growth advantage for cancer cells. Blocking of *O*-GlcNAcylated PFK1 led to a reduction of cancer cell proliferation *in vitro* and impaired tumor formation *in vivo*. Other glycolytic enzymes also reported to be modified by *O*-GlcNAc include triose phosphate isomerase (TPI) (21), glyceraldehyde-3-phosphate dehydrogenase (GAPDH) (22–25), enolase 2 (Eno2) (22, 25–27), and pyruvate kinase M2 (PKM2) (25). Although the glycosylation sites of these glycolytic enzymes have not been identified, their modifications could potentially modulate tumor cell metabolism promoting proliferation.

Glutamine is a major source of energy for rapidly dividing cells and is also an amino donor substrate for the conversion of fructose-6-phosphate to glucosamine-6-phosphate by GFAT in the HBP. Glutamine enters into cells via the glutamine transporter, which is found to be overexpressed in various cancers (28, 29). If GFAT is active, glutamine uptake in cancer cells can increase the HBP flux, as well as UDP-GlcNAc, and *O*-GlcNAcylation. This augmentation, for example, is found in human PDAC (30). In a related context, inhibition of glutaminase, an amidohydrolase enzyme, which generates glutamate from glutamine, led to a lower proliferation rate in human breast cancer cells (31). This inhibition also caused a reduction of GFAT activity and changes in *O*-GlcNAc targets, such as OGT and transcription factor Sp1. Moreover, removal of glutamine from the culture medium promotes tumor cell differentiation and decreased proliferation; conversely, addition of glutamine protects cells from apoptosis and induces proliferation (32). Thus, metabolic shifts through the HBP flux, hyperglycemia, glutamine consumption, elevation of UDP-GlcNAc, and *O*-GlcNAcylation contribute in regulating signaling cascades and cell proliferation in cancer.

## Regulation of *O*-GlcNAc Cycling Enzymes and *O*-GlcNAcylation

*O*-GlcNAcylation is tightly regulated by *O*-GlcNAc cycling enzymes, OGT and OGA for the addition and removal of sugar in and out of proteins, respectively. In human beings, there is only a single *OGT* gene localized at chromosome Xq13.1 (33). The alternative splicing of the *OGT* gene translates into at least three different isoforms of OGT enzyme, including the 110-kDa nucleocytoplasmic isoform (ncOGT), the 103-kDa mitochondrial isoform (mOGT), and the short 78-kDa isoform (sOGT) (34). The different isoforms of OGT differ in the number of tetra-tricopeptide repeats (TRP) located at the N-terminal domain, which is involved in protein interaction and is important for protein substrate recognition (33). OGT is inhibited by various inhibitors such as ST045849 (35). A single OGA encoding gene, identified as meningioma-expressed antigen 5 (*MGEA5*), is localized on chromosome 10q24.1–q24.3 (33). The two isoforms of OGA from alternative splicing have been identified (7). The 130-kDa isoform localizes predominantly in the cytoplasm, while the 75-kDa isoform, lacking one-third of the C-terminal domain, resides in the nucleus (33). The N-terminal domain of OGA is the catalytic domain, while the C-terminal domain contains a histone acetyltransferase (HATs) sequence (33). This enzyme can be inhibited with inhibitors such as PUGNAc, Thiamet G (36), and GlcNAcstatin (37). A number of studies have revealed complex formation between OGA and OGT. In a yeast-two hybrid screening study, OGA has been reported as a binding partner of OGT (38). The OGA-OGT complex has also been identified in combination with mSin3A, and histone deacetylase-1 (HDAC1) when the estrogen and progesterone signaling are stimulated in CHO cells (39). Therefore, it is possible that the binding between OGA and OGT may affect the regulation of each other under specific conditions.

*O*-GlcNAcylation in cancer has been increasingly studied for the past decade. Accumulating evidence reveals that the levels of *O*-GlcNAcylation and its cycling enzymes in malignant tissues are altered in various cancers. Recently, we showed that *O*-GlcNAcylation and OGT expression levels are increased in both breast and CRC (25, 40). As mentioned above, the *O*-GlcNAc cycling enzymes OGT and OGA tightly regulate the level of *O*-GlcNAcylation. Enhanced *O*-GlcNAcylation level corresponding to increased OGT and decreased OGA expression is commonly observed in various cancers including bone (41), bladder (42), breast (26, 43–45), bile duct (46), colon (47–49), leukemia (50), liver (51), lung (47), ovary (36), pancreas (30), and prostate (52, 53). Alteration of this glycosylation in thyroid cancer, however, occurs in the opposite way (54). Interestingly, no mutations in OGT and OGA genes have been reported in human cancers, suggesting that these enzymes are tightly conserved. Therefore, besides increased flux through the HBP, the altered expression of OGT and OGA also contributes to enhancement of *O*-GlcNAcylation in most cancers.

Manipulation and regulation of *O*-GlcNAc cycling enzymes in cancer may be a way of stopping cancer growth. We have shown that OGT silencing led to a reduction of anchorage-independent growth of a breast cancer cell line, MDA-MB-231 (25). Caldwell et al. also reported that reduction of OGT in breast cancer cells caused inhibition of tumor growth, both *in vitro* and *in vivo* (43). OGT knockdown did not block cell growth in a non-transformed breast cell line, MCF-10A (43). Consistent with this finding, reduction of *O*-GlcNAcylation had no effect on non-transformed pancreatic epithelial cell growth, but inhibited human PDAC cell proliferation and anchorage-independent growth, and triggered apoptosis (30). Because cancer cells appear to overexpress OGT, strategies to reduce OGT activity or expression level are attractive as an anti-cancer approach. Recently, the crystal structure of human OGT was solved by Lazarus et al. (55). As mentioned above, there is only one OGT gene in mammals with three alternative splicing isoforms, but more than one thousand *O*-GlcNAc proteins have been identified so far (dbOGAP: Database of *O*-GlcNAcylated Proteins and Sites). How does one OGT specifically modify a wide range of target proteins? OGT actually has protein-binding partners, which can form a transient complex under specific stimulation such as by nutrient, stress, and hormone. These partners include p38, OIP106, and OGA (38). The binding between OGT and its adaptor specifically targets the catalytic site of OGT to *O*-GlcNAcylate its target proteins (55). This knowledge will accelerate the rational design of OGT inhibitors for anti-cancer drug in the future.

## *O*-GlcNAc Modification and Phosphorylation in Cancer

*O*-GlcNAcylation has been studied widely for 30 years. Novel methodologies for enrichment of *O*-GlcNAc modified proteins are now available, as well as mass spectrometric methods for their characterization. Increasing study of *O*-GlcNAc proteins suggest extensive crosstalk between *O*-GlcNAcylation and phosphorylation. Crosstalk between these two modifications occurs not only by sharing their protein substrates but also by regulating each other’s cycling enzymes. Both post-translational modifications share many characteristics including the cycling of their substrates at a similar time scale, the site of modification, and cellular state (33). This becomes more complicated when several studies show *O*-GlcNAcylation of many kinases (56), as well as phosphorylation of OGT and OGA (10).

The *O*-GlcNAc attachment sites can be predicted using online software (57) such as Yin-Yang and dbOGAP (Database of *O*-GlcNAcylated Proteins and Sites). These programs can be used to determine the interplay between *O*-GlcNAcylation and phosphorylation. The crosstalk between *O*-GlcNAcylation and phosphorylation in cancer has been observed in various biological signaling regulators, including c-Myc (58, 59), p53 (57, 60), Snail1 (61), and NF-κB p65 subunit (30).

c-Myc is a transcription factor regulating transcription of many genes involved in cell proliferation, cell differentiation, and programed cell death, and displays a reciprocal interplay between both modifications. c-Myc at Thr-58 can be both a target for phosphorylation by GSK3 and *O*-GlcNAcylation (58, 59). Crosstalk between these two modifications is competitive depending on certain conditions. For example, *O*-GlcNAc modification at Thr-58 of c-Myc was higher than phosphorylation at the same site when cells are starved of serum. Serum stimulated cells showed the opposite result, since Thr-58 shows enhanced phosphorylation and decreased *O*-GlcNAcylation. Moreover, point mutation at Thr-58 in the coding region of c-*myc* is frequently found in human Burkitt lymphomas (59, 62). Therefore, modifications of this site might be crucial for tumor progression.

Yang et al. reported *O*-GlcNAc modification of p53 at Ser-149 in a breast cancer cell line, MCF-7 and a lung cancer cell line, H1299 (57, 60). *O*-GlcNAcylation at Ser-149 reduced phosphorylation at Thr-155, leading to disruption of binding between Mdm2 and p53, which consequently reduced p53 ubiquitin-proteasome degradation (57). Another study by Park et al. showed the important role of *O*-GlcNAcylation of Snail1, a transcriptional repressor of E-cadherin (61). Snail1 is phosphorylated by GSK-3β, promoting its ubiquitination and degradation (63). Elevated *O*-GlcNAc level caused by OGA inhibitors inhibited the phosphorylation-mediated proteasomal degradation of Snail1 and consequently increased Snail1 half-life in a similar manner to p53.

Nuclear factor-kappa B (NF-κB) is a well-known transcription factor regulating cytokine production, lymphocyte activation, and proliferation. NF-κB activation was found in lymphoma and many solid tumors (64). NF-κB is a dimer of p65 (RelA) and p50 subunits. Ma et al. reported *O*-GlcNAc modification of NF-κB p65 subunit and IKKα/IKKβ in human PDAC (30). Phosphorylation of the p65 subunit was increased when global *O*-GlcNAcylation was reduced.

Crosstalk between *O*-GlcNAcylation and phosphorylation in cancer are not always reciprocal. Examples include vimentin and heat shock protein 27 (HSP27), as well as keratin 8 and 18. *O*-GlcNAc sites of vimentin are on Ser-7, Thr-33, Ser-34, and Ser-54 (65). Alteration of *O*-GlcNAc level by OGA overexpression in HeLa cells led to decreased pSer-82 level and increased pSer-71 level in vimentin (66). Guo et al. also showed that nuclear translocation of HSP27 observed in liver cancer cells is regulated by both *O*-GlcNAc and phosphate groups (67). Another study from Srikanth et al. displayed the synergistic effects of *O*-GlcNAcylation and phosphorylation on keratin 8 and 18 (68). The more *O*-GlcNAc modification occurs, the more phosphorylation was observed in soluble keratins compared to filamentous form. Moreover, increased *O*-GlcNAcylation and phosphorylation of keratin 8 and 18 were observed in heat stress-induced HepG2 cells.

## Colorectal Cancer and *O*-GlcNAcylation

### The expression levels of *O*-GlcNAc, OGT, and OGA in CRC

Colorectal cancer is one of the most common cancers worldwide. It ranks the third in men and second in women according to World Health Organization GLOBOCAN database, in 2012 (69). Even though CRC is a curable cancer, its mortality rate is remarkably high, accounting for 8% of all cancer deaths. Many studies are focused on molecular targets of CRC for finding biomarkers and improving treatments, but there is a little research on the regulation of *O*-GlcNAc, OGT, and OGA in CRC.

Significantly elevated levels of OGT and *O*-GlcNAcylation were observed in CRC tissues compared to adjacent normal tissues (47). However, OGA level was not significantly enhanced in such cancer tissue samples (47). Consistent with this finding, Phueaouan et al. also reported that, in primary CRC patients (grade II), the upregulation of *O*-GlcNAcylation and OGT enzyme was found in CRC tissues, but the expression of OGA did not differ in CRC tissue extracts compared to normal samples (40).

Two studies reported changes in global *O*-GlcNAcylation of CRC cell lines in association with biological effects. A colorectal adenocarcinoma cell line, HT29 was transfected with the sh*OGT* expressing lentiviral vector in order to knockdown the *OGT* gene (47). The level of *O*-GlcNAcylation was decreased, but this did not reduce invasion in HT29, but did diminish anchorage-independent growth. Increasing *O*-GlcNAc levels in Thiamet-G treated HT29 cells markedly enhanced colony formation in soft agar. Another study on the association of *O*-GlcNAcylation and CRC recently indicated that the metastatic SW620 clone showed higher *O*-GlcNAcylation level than the primary SW480 clone (48). Enhanced *O*-GlcNAcylation by si*OGA* knockdown in SW620 resulted in the alteration of morphology to a fibroblast-like morphology, associated with the epithelial metastatic progression, and growth retardation. In addition, transcriptomics by microarray analysis revealed that silencing of *OGA* can affect the expression of many genes involved in cell movement and growth, as well as in lipid and carbohydrate metabolism (48).

### *O*-GlcNAcylated proteins and O-GlcNAc targeting in CRC

**Figure 2:**
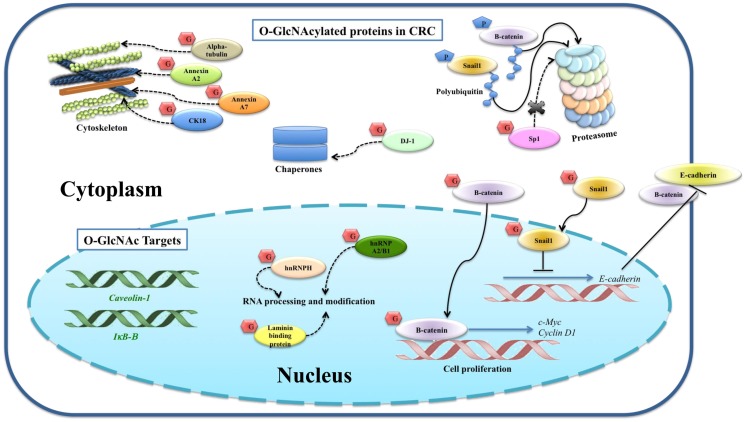
***O*-GlcNAcylated proteins and their targets identified in colorectal cancer**. *O*-GlcNAcylation stabilizes β-catenin and subsequently translocates into the nucleus for its gene activation. *O*-GlcNAcylation stabilizes Snail1, which subsequently represses E-cadherin expression level observed in other cancer cells, suggesting a proposed mechanism for colorectal cancer. Phosphorylation of β-catenin and Snail1 is proposed to activate proteasomal degradation. The proposed mechanisms for other *O*-GlcNAcylated proteins, including SP1, CK18, α-tubulin, hnRNPA2/B1, hnRNPH, annexin A2, annexin A7, laminin-binding protein, and protein DJ-1, are also indicated. Expression levels of *E-cadherin, β-catenin, caveolin-1*, and *IκB-β* are altered corresponding to increased global *O*-GlcNAcylation, so they are categorized as *O*-GlcNAc downstream targets. Solid lines indicate known mechanisms, whereas dashed lines are proposed mechanisms.

A multistage carcinogenesis model of CRC progression was proposed by Vogelstein et al. (70). The initial step, which changes normal epithelium cells to early adenoma, includes mutation of *APC* (adenomatous polyposis coli) gene resulting in nuclear accumulation of protooncogene β-catenin (49). Nuclear β-catenin activates the transcription of c-Myc and cyclin D1, which are important for cell proliferation. β-Catenin is negatively regulated by phosphorylation leading to proteasomal degradation. Mutation of β-catenin at specific amino acids stabilizes β-catenin and subsequent nuclear localization (71). Little information has been reported on *O*-GlcNAc modified proteins in CRC (Figure 2). *O*-GlcNAcylated proteins were identified in the CRC molecular signaling pathway, including β-catenin (49) and Snail1 (61). Olivier-Van Stichelen et al. found that β-catenin of the normal colon cell line, CCD841CoN, showed less *O*-GlcNAcylation compared to two other CRC cells, HT29 and HCT116 (72). Other work from the same group demonstrated that β-catenin and global *O*-GlcNAc levels were increased in proteins extracted from colons of mice fed with high carbohydrate diet and Thiamet G (49). Four *O*-GlcNAcylation sites on β-catenin including Ser-23, Thr-40, Thr-41, and Thr-112 were mapped by ETD-MS/MS. Increased global *O*-GlcNAcylation of CRC cells reduced phosphorylation of β-catenin at Thr-41, located in the D box of β-catenin, which is important for proteasomal degradation.

Snail1, a transcriptional suppressor of E-cadherin, is upregulated in CRC tissues (73, 74). E-cadherin functions in cell adhesion, which is required for cell differentiation and homeostasis of epithelium. Suppression of E-cadherin accelerates invasion and is associated with a more malignant phenotype and poor differentiation in CRC (75). Snail1 was pulled down using sWGA affinity from SW480 CRC cell (61), and although *O*-GlcNAcylated Snail1 function was not studied in this cell line, its role was proposed according to data from HEK293 and A549 cells. *O*-GlcNAcylation at Ser-112 of Snail1 has been mapped in HEK293 cell (61). As mentioned earlier, Ser-112 is crucial for phosphorylation-mediated proteasomal degradation of Snail1. *O*-GlcNAc modification can stabilize Snail1 and subsequently inhibit mRNA expression levels of E-cadherin. Hyperglycemia also induced *O*-GlcNAcylation of Snail1 and suppressed E-cadherin expression level, resulting in stimulation of epithelial–mesenchymal transition (EMT).

Sp1, a specificity protein 1 transcription factor, regulates various genes encoding for growth factors, receptors, and proteins involved in cell growth, apoptosis, differentiation, and immune responses (76). *O*-GlcNAcylation of immunoprecipitated Sp1 in HT29 cell was reported by Haltiwanger et al. (77). Increased *O*-GlcNAcylation by treatment with PUGNAc showed a reciprocal effect to phosphorylation on Sp1. However, the consequence of *O*-GlcNAcylation of Sp1 in HT29 cell was not reported. Interestingly, other works showed the function of *O*-GlcNAcylation in protecting Sp1 from the ubiquitin-proteasome pathway (76, 78).

Gel based proteomics was used by our group to study the *O*-GlcNAc profile in CRC tissues (40). Eight *O*-GlcNAc modified proteins showed an increase in *O*-GlcNAcylation including cytokeratin 18 (CK18), α-tubulin, heterogeneous nuclear ribonucleoproteins (hnRNPs) A2/B1 (hnRNPA2/B1), hnRNPH, annexin A2, annexin A7, laminin-binding protein, and protein DJ-1 (40). CK18 was reported to be modified by *O*-GlcNAc in at least three sites (Ser-30/Ser-31/Ser-49) (79). Increased *O*-GlcNAc-CK18 was associated with increased solubility and decreased cellular levels, while absence of *O*-GlcNAc on CK18 increased stability (68). α-tubulin was also identified as an *O-*GlcNAc modified protein by Walgren et al. (80). Increased *O*-GlcNAc α-tubulin resulted in a reduced hetero-dimerization into microtubules (81). Annexin A2 and A7 play important roles in cytoskeletal formation and cell matrix interaction. *O*-GlcNAcylation of annexin A2 was found to be overexpressed in all cancer samples (7/7) (40). HnRNPA2/B1 and H are a group of RNA-binding proteins involved in various processes in RNA metabolism including pre-mRNA splicing, mRNA transport, and translation. Protein DJ-1 plays a role as antioxidant and/or a molecular chaperone. Laminin-binding protein is involved in the assembly and/or stability of the ribosome in the nucleus. Although the sites of *O*-GlcNAcylation of such proteins have not been mapped in CRC tissues, the results presented here showed detectable *O*-GlcNAc modified proteins in clinical samples, which are promising as novel potential CRC biomarkers.

Furthermore, *O*-GlcNAcylation affects expression levels of a number of genes in the SW620 cell (48). Silencing of *OGA* affected the expression of about 1300 genes associated with cell movement and growth, as well as metabolic pathways involving lipids and carbohydrates. Among these, E-cadherin, β-catenin, and caveolin-1 proteins were upregulated, while IκB-β was downregulated when the cell was transfected by *siOGA* or Thiamet-G treatment, respectively. This also suggests that alteration of *O*-GlcNAcylation plays a vital role in the regulation of gene expression in CRC.

## Breast Cancer and *O*-GlcNAcylation

### The expression levels of *O*-GlcNAc, OGT, and OGA in breast cancer

Breast cancer is the most frequently observed cancer among women with an estimated 1.67 million new cancer cases diagnosed in 2012 (25% of all cancers). Moreover, breast cancer ranks as the fifth cause of death from cancer overall (69). Many women who develop breast cancer have no obvious risk factors for breast cancer. Since intervention cannot always guarantee prevention of breast cancer, more research is required for identification and development of early stage biomarkers and molecular targets for effective drug treatment.

Several studies of *O*-GlcNAc and its cycling enzyme expression have been studied in breast cancer. In 2001, Slawson et al. found that *O*-GlcNAcylation was decreased and this is due to an increase in hexosaminidase and OGA activity in primary breast tumors, compared to matched normal adjacent breast tissues (82). However, Dahl et al. reported a gene named *MGEA5* coding for OGA, which showed about 56% reduction in expression in breast cancer tissues (83). Several groups, including Gu et al. (44), Caldwell et al. (43), Krzeslak et al. (84), and our group (25), showed that the expression of *O*-GlcNAcylation and OGT are upregulated, while the OGA expression is downregulated in breast cancer. Manipulation of OGT and OGA activities in breast cancer cells showed that overexpression of OGT enhanced the migration/invasion of breast cancer cells *in vitro* and lung metastasis *in vivo*, but did not affect cell proliferation (44). Conversely, reduction of *O*-GlcNAcylation through RNA interference of OGT in breast cancer cells led to inhibition of tumor growth both *in vitro* and *in vivo* (43). Similarly, OGT silencing resulted in a reduction of anchorage-independent growth of a breast cancer cell line, MDA-MB-231 (25). Recently, increased *O*-GlcNAcylation level was found to protect the breast cancer cell line, MCF-7 from tamoxifen induced cell death, whereas siRNA mediated OGT knockdown had opposite effects (85). These data suggest that OGT may represent a novel therapeutic target of cancer, especially in overcoming tamoxifen resistance in breast cancer.

### *O*-GlcNAcylated proteins and *O*-GlcNAc targeting in breast cancer

**Figure 3:**
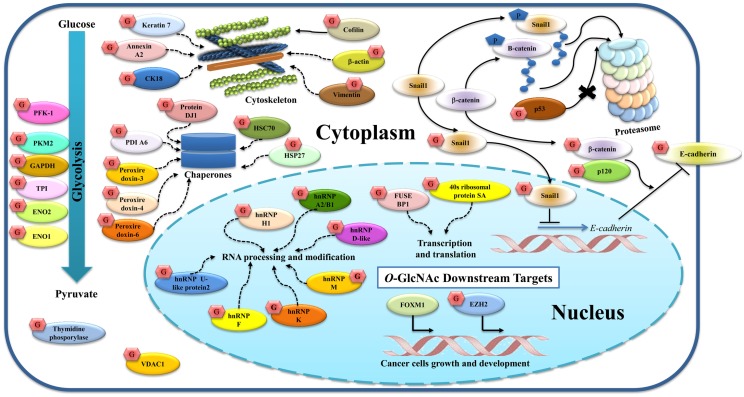
***O*-GlcNAcylated proteins and their targets identified in breast cancer**. *O*-GlcNAcylation modifies many proteins in both the cytoplasm and nucleus. p120, β-catenin, and E-cadherin are glycosylated, and this modification regulates E-cadherin localization and stability. *O*-GlcNAc Snail1 suppresses E-cadherin expression. Phosphorylation of β-catenin and Snail1 can activate proteasomal degradation while *O*-GlcNAc p53 protects this event. Other cytoskeletal proteins (cofilin, β-actin, vimentin, keratin 7, annexin A2, and CK18), glycolytic enzymes (PKF1, PKM2, GAPDH, TPI, ENO2, and ENO1), chaperones (protein DJ1, HSC70, HSP27, PDIA6, peroxiredoxin 3, 4, and 6), thymidine phosphorylase, and VDAC1 are also *O*-GlcNAcylated. Nuclear proteins including hnRNPs and proteins related to transcription and translation are *O*-GlcNAcylated as indicated. Alteration of this modification also regulates gene and protein expressions in breast cancer including FoxM1 and EZH2. Solid lines indicate known mechanisms, whereas dashed lines are proposed mechanisms.

Many groups of researchers have reported *O*-GlcNAc targets in breast cancer (Figure 3).Growing evidence suggests that *O*-GlcNAc plays vital roles in the regulation of cellular adhesion and cytoskeletal formation. Gu et al. showed that *O*-GlcNAcylation of p120 and β-catenin played roles in decreasing the level of E-cadherin at the cell surface (44). E-cadherin was also reported to be modified by *O*-GlcNAc at its cytoplasmic domain in breast cancer (86). Later, Geng et al. showed that *O*-GlcNAcylated E-cadherin interferes with the binding of Type I gamma phosphatidylinositol phosphate kinase (PIPKIγ), a protein required for recruitment of E-cadherin to adhesion sites, leading to reduced E-cadherin trafficking to the plasma membrane and accelerated apoptosis (87). Other *O*-GlcNAcylated proteins found in breast cancer and described earlier in terms of their function are p53 (60) and Snail1 (61).

In a related context, cofilin, a family of actin-binding proteins, which disassembles actin filaments, has also been reported to be modified by *O*-GlcNAc at Ser-108 and this glycosylation is essential for invadopodia formation, a process involving extracellular matrix (ECM) degradation during cancer invasion and metastasis (88). Decrease in its *O*-GlcNAcylation leads to the destabilization of invadopodia and impairs the invasion of breast cancer cells. Komura et al. reported that GlcNAc polymers and *O*-GlcNAc proteins induce the expression of vimentin and cell migration in MCF-7 (89). Regulation of vimentin expression by GlcNAc may play a crucial role for the EMT. We also showed that vimentin, cytokeratin 18 (CK18), β-actin, and keratin 7 appeared to show increased *O*-GlcNAcylation in breast cancer tissues, but their glycosylation sites were not yet mapped (25).

Glycolytic enzymes are also targets of *O*-GlcNAcylation. Several glycolytic enzymes were hyper-*O*-GlcNAcylated in breast cancer tissues. Using *O*-GlcNAc gel based proteomics, four glycolytic enzymes including enolase 2 (ENO2), TPI, pyruvate kinase M2 (PKM2), and GAPDH were identified (25). *O*-GlcNAcylation of enolase 1 (ENO1) was also reported in a breast cancer cell line, T47D (26), but the glycosylation sites have not yet been identified. Interestingly, as described earlier, *O*-GlcNAc at Ser-529 of phosphofructokinase 1 (PFK1) inhibited its activity and redirected glucose flux through the PPP (20). Recently, Ferrer et al. demonstrated that *O*-GlcNAcylation regulates glycolysis in cancer cells via hypoxia-inducible factor 1 (HIF-1α) and glucose transporter type 1, GLUT1 (90). Reducing *O*-GlcNAcylation led to HIF-1α degradation and activation of ER stress and apoptosis. In addition, human breast cancers with high HIF-1α and OGT levels, and low OGA levels are correlated with poor patient outcome. This suggests that the combined detection of HIF-1α, OGT, and OGA in clinical samples may be useful as potential breast cancer biomarkers.

Heterogeneous nuclear ribonucleoproteins are also a major group of proteins modified by *O*-GlcNAc in breast cancer. The hnRNPs are complexes of RNA and proteins that are involved in multiple aspects of RNA processing and modifications. Five members of hnRNPs including hnRNP U-like protein 2, hnRNPK, hnRNPF, hnRNPM, and hnRNPA2/B1 showed increased *O*-GlcNAcylation in breast cancer tissues (25), while other work by Rambaruth et al. showed that hnRNP H1, hnRNP D-like, hnRNP A2/B1 were hyper-*O*-GlcNAcylated in breast cancer cells (26). Although their glycosylation sites were not identified, increased *O*-GlcNAcylation in the hnRNP family may act as a novel regulation of alternative mRNA processing and gene expression, and that promotes a beneficial phenotype for cancer. In addition, two proteins in the nucleus involved in transcription and translation, ribosomal protein SA (RSSA) and far upstream element-binding protein 1 (FUSE-BP1), showed increased *O*-GlcNAcylation in cancer (25).

Chaperones and stress response proteins also show *O*-GlcNAc modification. We reported that heat shock cognate proteins (HSC70), protein disulfide isomerase (PDI) A6, peroxiredoxin 3, 4, and 6, and protein DJ-1 are *O*-GlcNAcylated in breast cancer tissues (25), while Rambaruth et al. showed *O*-GlcNAcyation of HSP27 (26). However, the effect of this modification on chaperones and stress response function is still not clear.

Two other proteins may be hyper *O*-GlcNAcylated: thymidine phosphorylase (TP), an enzyme involved in nucleic acid metabolism, and voltage dependent anion selective channel protein 1 (VDAC1), a mitochondrial protein that may contribute in triggering apoptosis (25).

*O*-GlcNAcylation also regulates gene and protein expression in breast cancer. Caldwell et al. showed that decreasing *O*-GlcNAcylation using RNA interference against OGT led to decreased cell invasion, tumor growth, and angiogenesis, both *in vitro* and *in vivo*, and this reduction is associated with decreased expression and activity of the oncogenic transcription factor FoxM1 (43). Recently, Chu et al. showed that the enhancer of zeste homolog 2 (EZH2), an enzyme, which acts as a gene silencer by histone methylation, is *O*-GlcNAcylated at Ser-75 (91). OGT knockdown reduced the EZH2 expression and H3 trimethylation at K-27 in MCF-7, indicating that *O*-GlcNAcylation of EZH2 is required for EZH2 stability.

## Conclusion and Perspectives

Current information strongly suggests that alteration of cellular metabolism from mitochondrial oxidative phosphorylation to aerobic glycolysis provides both bioenergetics and biosynthesis capability for cancer cells. Increasing glucose uptake and the redirection of glucose to the HBP flux can lead to an increase of UDP-GlcNAc and *O*-GlcNAcylation levels in cancer. Mutation of genes involving glucose uptake also contributes to this. In addition, *O*-GlcNAc cycling enzymes are altered in most cancers. Changes in *O*-GlcNAc and OGT levels clearly show positive relationship with the histological grade of breast and colorectal tumors. Greater increases in *O*-GlcNAc levels correlated with higher grades of tumor development. Importantly, this modification has extensive crosstalk with phosphorylation, which consequently affects cellular signaling. Thus, metabolic shifts through the HBP flux and *O*-GlcNAcylation contribute to regulation of signaling cascades and cell proliferation in cancer.

Study of *O*-GlcNAcylation in cancer is rapidly growing in terms of both detection and functional studies using *in vitro* and *in vivo* models. Several *O*-GlcNAc modified proteins have been discovered in breast and CRC. However, more *O*-GlcNAcylated proteins have been identified in breast cancer in comparison with CRC. This may result from differences in protein expression in each specific organ, as well as the tumor grade examined in the study. In addition, the levels of *O*-GlcNAc cycling enzymes differ. In breast cancer, major identified proteins are glycolytic enzymes and proteins, which function in biosynthesis (nucleic acid metabolism). The modified protein groups shared in breast and CRC are (1) proteins, which function in the stress responses; (2) hnRNPs and proteins involved in transcription and translation; (3) proteins related to the cytoskeleton and their regulation; and (4) transcription factors (e.g., Snail1 and β-catenin), which are difficult to observe and need to be enriched before detection. In addition, a number of *O*-GlcNAc targets in breast and CRC are not shown to be modified directly but rather regulate gene and/or protein expression such as FoxM1, Caveolin-1, and IκB-β. Moreover, more *O*-GlcNAc modified proteins were identified from breast cancer tissues than from breast cancer cell lines, indicating the complexity of *O*-GlcNAc regulation *in vivo* (25). *O*-GlcNAc modified proteins identified from clinical samples are thus more realistic as potential novel cancer biomarkers. Examples are PKM2 in breast cancer (25) and annexin A2 in CRC (40). However, larger scale studies need to be performed to obtain information with greater accuracy and reliability for possible use in clinical detection. On another hand, *O*-GlcNAc research on cell lines is also needed in order to test mechanisms and functions. A good example is that of *O*-GlcNAcylation of PFK1, which leads to decrease in activity and redirection of glucose metabolism in cancer cell lines (20). Alteration of OGT expression both in breast cancer cells (*in vitro*) and in animal model (*in vivo*) also suggests a promising approach for anti-cancer therapy (43). Research from both specimen samples and cell lines is, therefore, needed to provide a better understanding of *O*-GlcNAc biology in cancer. Taken together, this review shows the current findings on *O*-GlcNAcylation in breast and CRC. Many *O*-GlcNAc modified proteins are promising as potential novel cancer biomarkers or may be used in combination with standard detection (e.g., serum biomarkers) to enhance specificity and accuracy. Ongoing research will aim to detect these modified proteins in large-scale samples specifically and rapidly. Many detection techniques such as advanced mass analyzers with new fragmentation techniques including electron transfer dissociation (ETD) are being developed. Specific *O*-GlcNAc modified proteins in cancer specimens, therefore, are challenging to discover as potential candidates for cancer diagnosis, especially in breast and CRC.

## Conflict of Interest Statement

The authors declare that the research was conducted in the absence of any commercial or financial relationships that could be construed as a potential conflict of interest.
